# Host-versus-commensal immune responses participate in the rejection of colonized solid organ transplants

**DOI:** 10.1172/JCI153403

**Published:** 2022-09-01

**Authors:** Isabella Pirozzolo, Martin Sepulveda, Luqiu Chen, Ying Wang, Yuk Man Lei, Zhipeng Li, Rena Li, Husain Sattar, Betty Theriault, Yasmine Belkaid, Anita S. Chong, Maria-Luisa Alegre

**Affiliations:** 1 Department of Medicine, Section of Rheumatology, University of Chicago, Chicago, Illinois, USA.; 2 Columbia University Vagelos College of Physicians and Surgeons, New York, New York, USA.; 3 Exelixis, Alameda, California, USA.; 4 Department of Pathology and; 5 Department of Surgery, University of Chicago, Chicago, Illinois, USA.; 6 National Institutes of Health, Bethesda, Maryland, USA.

**Keywords:** Immunology, Transplantation, Adaptive immunity, Memory

## Abstract

Solid organ transplantation is the preferred treatment for end-stage organ failure. Although transplant recipients take life-long immunosuppressive drugs, a substantial percentage of them still reject their allografts. Strikingly, barrier organs colonized with microbiota have significantly shorter half-lives than non-barrier transplanted organs, even in immunosuppressed hosts. We previously demonstrated that skin allografts monocolonized with the common human commensal *Staphylococcus epidermidis* (*S.epi*) are rejected faster than germ-free (GF) allografts in mice because the presence of *S.epi* augments the effector alloimmune response locally in the graft. Here, we tested whether host immune responses against graft-resident commensal microbes, including *S.epi*, can damage colonized grafts independently from the alloresponse. Naive hosts mounted an anticommensal T cell response to colonized, but not GF, syngeneic skin grafts. Whereas naive antigraft commensal T cells modestly damaged colonized syngeneic skin grafts, hosts with prior anticommensal T cell memory mounted a post-transplant immune response against graft-resident commensals that significantly damaged colonized, syngeneic skin grafts. Importantly, allograft recipients harboring this host-versus-commensal immune response resisted immunosuppression. The dual effects of host-versus-commensal and host-versus-allograft responses may partially explain why colonized organs have poorer outcomes than sterile organs in the clinic.

## Introduction

Solid organ transplantation is the preferred treatment for end-stage organ failure. Recipients of allogeneic grafts mount immune responses that can lead to transplant rejection, even when hosts are treated with immunosuppressive drugs. Importantly, the survival of grafts depends on the type of organ transplanted; within 5 years of surgery, 41% of lung and 54% of intestinal transplant recipients were reported to have rejected their grafts (1). In that same time frame, only 27% rejected a kidney and 23% a heart (1–3). Several factors distinguish these tissue types. One critical differentiator is the presence of donor microbiota on organs with the shortest half-lives and the absence of donor microbiota on organs that survive longer. The microbiota is vastly different between distinct individuals (4), raising the possibility that hosts of colonized organ allografts mount an immune response not only to mammalian cells within the graft, but also to donor commensals that accompany the tissue.

In previous work, we demonstrated that the presence of a single commensal organism, *Staphylococcus epidermidis* (*S.epi*), on a skin allograft was sufficient to accelerate allograft rejection in comparison with germ-free (GF) transplants. *S.epi* augmented the expression of inflammatory cytokines in the skin allograft and enhanced the effector phase of the host’s alloresponse locally in the graft (5). This published study investigated *S.epi*’s impact on the alloresponse but did not address whether a concurrent immune response to *S*.*epi* was elicited and, if so, whether it contributed to transplant rejection.

Patients, like conventional mice housed in specific pathogen–free (SPF) conditions, harbor a complex microbiota that generates a diverse repertoire of commensal-specific lymphocytes (4, 6, 7). Many of these lymphocytes are tissue-resident memory T cells induced upon colonization with specific commensals (8–10). These cells are poised to recognize commensal organisms at steady state and respond in a proinflammatory manner following tissue injury (11–13). Our results show that commensal-specific host naive and memory T cells mount an anticommensal immune response following transplantation with colonized organs. This host-versus-commensal immune response acts in parallel to the host-versus-graft alloresponse and causes hosts to resist immunosuppression, thus providing a mechanistic explanation for why colonized organs have a shorter half-life than sterile organs following transplantation.

## Results

### Skin graft recipients mount a T cell response to commensals on donor organs that is independent from the alloresponse.

**Figure 1:**
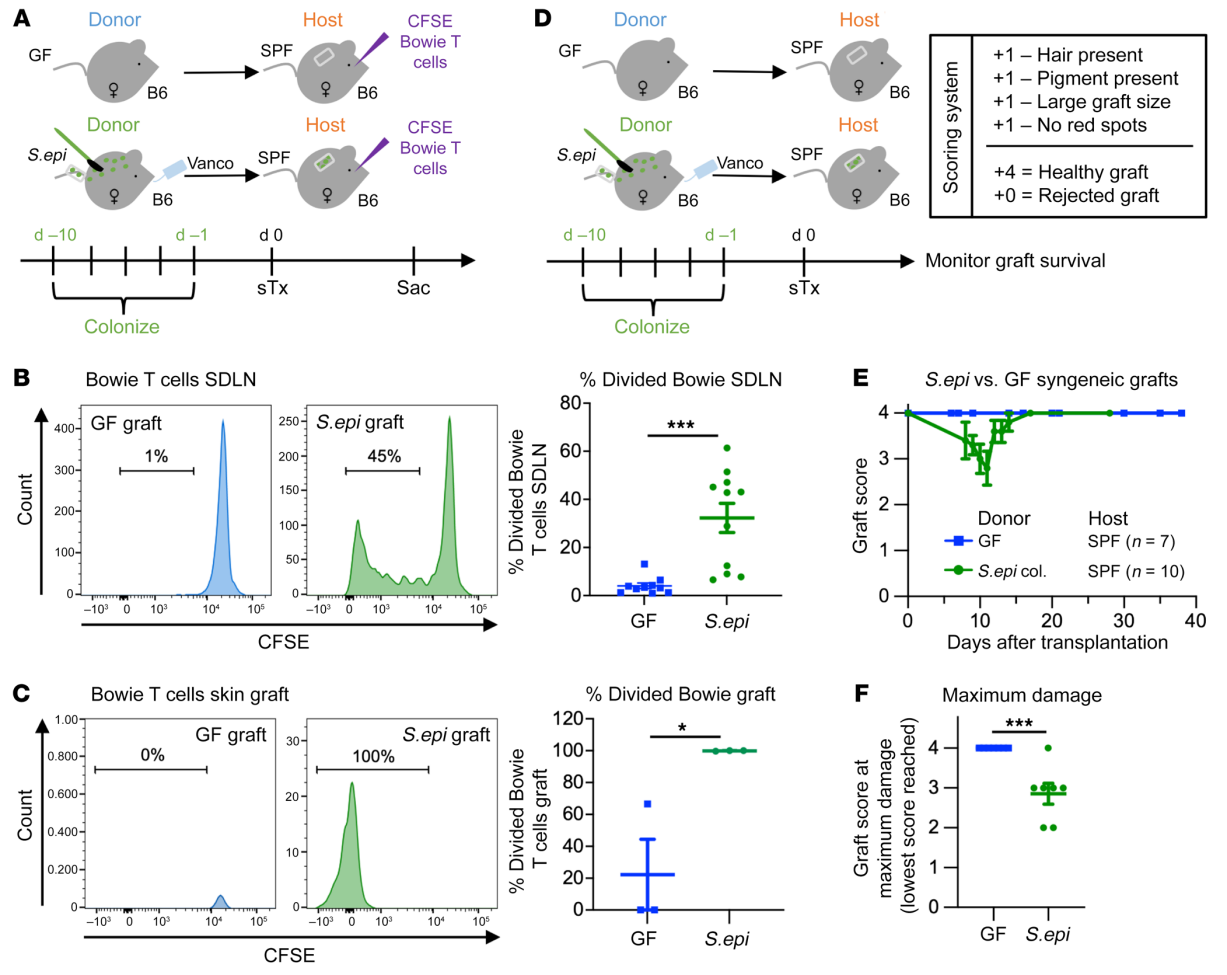
Skin graft recipients mount a T cell response to commensals on syngeneic donor organs. (**A**) Skin graft recipients were seeded with CFSE-labeled *S.epi*-specific CD8^+^ TCR-transgenic Bowie T cells (1 × 10^6^ cells) and then given a GF or *S.epi*-monocolonized syngeneic skin graft. B6, C57BL/6. (**B** and **C**) CFSE dilution of Bowie cells was examined 6 days (**B**) and 10 days (**C**) after transplantation in the SDLNs (**B**) and skin graft (**C**). Plots represent mean ± SEM and were analyzed by Welch’s unpaired, 2-tailed *t* test. Results were pooled from three (**B**) independent experiments. (**D** and **E**) *S.epi*-monocolonized or GF skin grafts were transplanted onto syngeneic SPF mice. Grafts were scored on a 4-point scale, gaining 1 point each for the presence of hair, the presence of pigment, a large graft size, and the absence of red spots. (**E**) These scores were plotted over time. (**F**) The lowest graft score each individual graft reached was plotted. Scores were analyzed using an unpaired, 2-tailed *t* test. *P < 0.05, ***P < 0.0005; col., colonized; d, day.

Organs colonized with commensal microbiota have a shorter half-life following transplantation than sterile organs. Using a mouse model of allogeneic skin transplantation, we previously demonstrated that one mechanism explaining why rejection of a skin allograft colonized with *S.epi* is faster than rejection of a GF skin allograft is that cutaneous *S.epi* augments the effector phase of the alloresponse, locally, when alloreactive T cells enter the skin allograft (5). We reasoned that another way donor skin colonization might accelerate graft loss is by prompting the host to mount a specific immune response to the donor commensals present in the graft. This response would accelerate graft loss if it contributed to post-transplant graft damage. We adopted the skin colonization regimen used previously (5) in which GF donor mice were painted with the common human skin commensal *S.epi* (strain NIHLM087), every other day for 10 days. To ensure that colonization was restricted to the skin, animals received vancomycin in their drinking water to eliminate *S.epi* that may have entered their intestinal tract during grooming (Figure 1A). This protocol resulted in colonization of the donor’s skin, and not the intestine, for at least 2 weeks after the last painting (Supplemental Figure 1, A–C; supplemental material available online with this article; https://doi.org/10.1172/JCI153403DS1).

To investigate whether the host mounted an anti–donor commensal immune response following skin transplantation in the absence of a concurrent alloresponse, we used syngeneic transplants, and *S.epi*-specific CD8^+^ TCR-transgenic T cells (Bowie T cells) to track anti-*S.epi* T cell responses. Skin grafts from female GF or *S.epi*-colonized mice were placed onto SPF syngeneic female hosts that had been seeded with CFSE-labeled Bowie T cells 1 day before transplantation. Hosts were sacrificed 6 days after transplantation (Figure 1A). Bowie T cells in the host’s skin graft–draining lymph nodes (SDLNs) proliferated (Figure 1B) and upregulated the marker of antigen experience CD44 (Supplemental Figure 2, A and B) in mice that received *S.epi*-colonized, but not GF, skin grafts. By 10 days after transplantation, divided CD44^hi^ Bowie T cells in recipients of colonized but not GF syngeneic grafts were detected in the graft itself (Figure 1C and Supplemental Figure 2, A and C). Activation of Bowie T cells in hosts of *S.epi*-colonized graft was driven by cognate antigen recognition, since, when hosts were cotransferred with *S.epi*-reactive Bowie T cells and irrelevant ovalbumin-reactive CD8^+^ OT I T cells, only Bowie but not OT I T cells expanded following transplantation of a *S.epi*-colonized skin graft (Supplemental Figure 2, A, D, and E). We confirmed by PCR that hosts housed in our SPF mouse facilities were not colonized with *S.epi* before transplantation, consistent with the fact that *S.epi* is not a native mouse commensal (Supplemental Figure 3A) (14). Notably, the surgical trauma of skin transplantation was not necessary to elicit an anticommensal response in the host, as Bowie T cells proliferated significantly, albeit modestly, in SPF mice without transplants following painting with *S.epi* (Supplemental Figure 3, B and C).

We next asked whether an endogenous T cell response against graft-resident commensals was sufficient to damage or reject syngeneic organs. To this end, we transplanted skin from GF or *S.epi*-colonized female mice into SPF syngeneic female hosts and monitored graft survival. We scored skin grafts on a 4-point scale for signs of chronic rejection. Grafts received 1 point each for the presence of hair, the presence of pigment, a large graft size, and an absence of red inflamed spots (Figure 1D). Brief and modest damage was observed on *S.epi*-colonized but not GF syngeneic grafts (Figure 1, E and F), with *S.epi*-colonized grafts displaying significantly more damage at their worst scoring point than GF grafts (Figure 1F). The damage was not due to infection, as there was no sign of erythema, purulence, or drainage at the time of bandage removal (day 7 after transplantation) (Supplemental Figure 4).

To address whether a higher frequency of *S.epi*-reactive naive T cells would heighten the damage to *S.epi*-colonized skin grafts, hosts received Bowie T cells (1.5 × 10^6^) 1 day before transplantation with a *S.epi*-colonized syngeneic skin graft (Supplemental Figure 5). Increasing the precursor frequency of *S.epi*-reactive naive T cells to supraphysiological levels did not worsen the damage in colonized syngeneic skin grafts. This could be because the endogenous response was already maximal or because Bowie T cells cannot cause skin graft damage. Nonetheless, the data indicate that a primary endogenous immune response against *S.epi* only modestly damages *S.epi*-colonized skin grafts.

### Hosts with memory against commensals present on donated organs display strong damage of syngeneic skin grafts.

**Figure 2:**
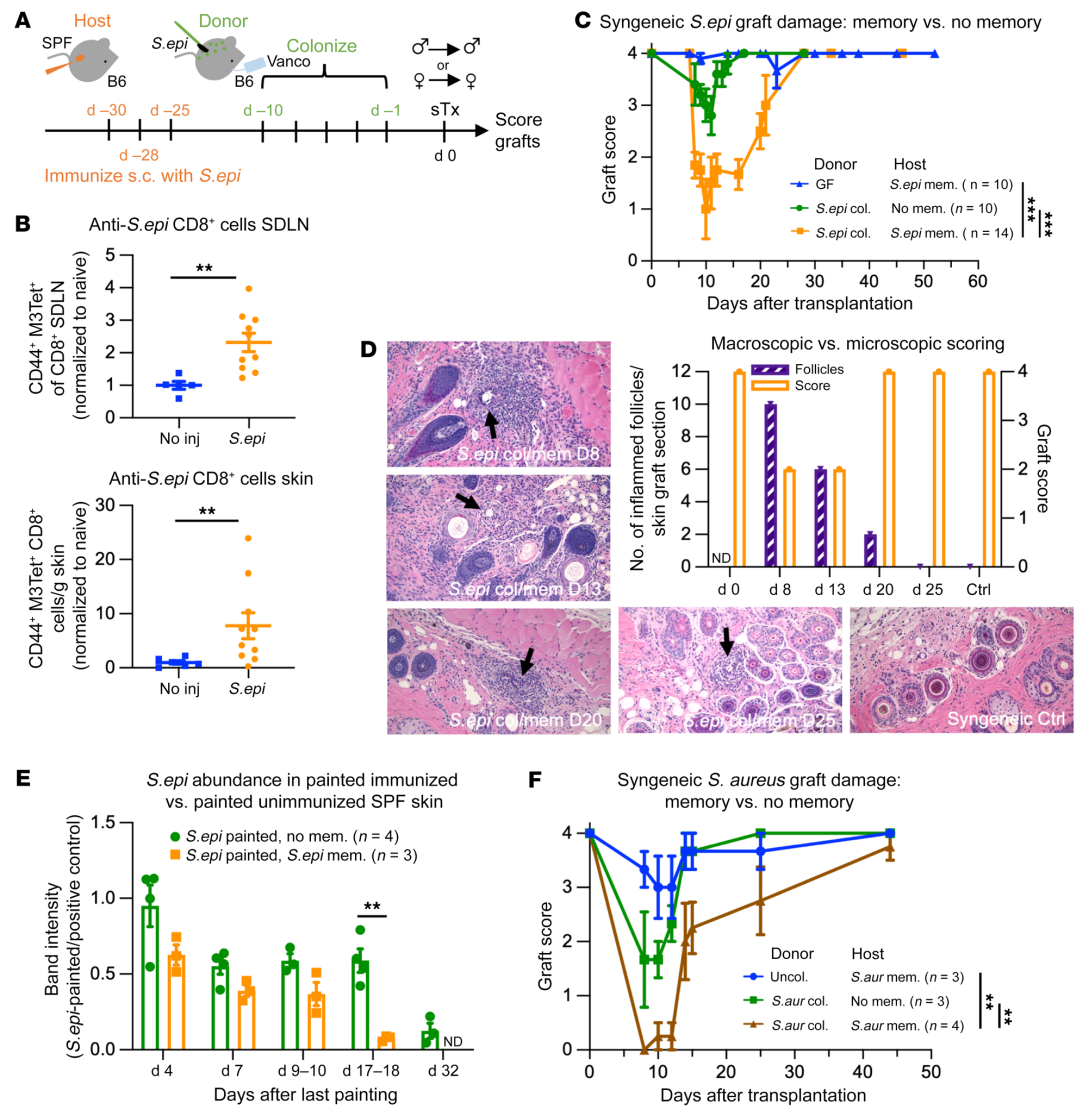
Hosts with memory against commensals present on donated organs damage colonized syngeneic skin grafts. (**A**) Hosts were injected with *S.epi* (1 × 10^5^ CFU s.c.) 30, 28, and 25 days before receiving *S.epi*-monocolonized syngeneic skin grafts. Grafts were scored on a 4-point scale (see Figure 1D). (**B**) Endogenous *S.epi*-specific T cells in the skin and SDLNs 1 month after *S.epi* immunization. Gated on CD45.2^+^TCRβ^+^CD8^+^CD4^–^CD44^+^ H2-M3:fMIIINA–PE^+^ (tetramer against *S.epi*-reactive T cells) events. Plots show fold change relative to unimmunized mice (mean ± SEM) analyzed by Wilcoxon’s log-rank test. (**C**) Graft scores of syngeneic hosts with or without anti-*S.epi* memory that received GF or *S.epi*-monocolonized skin grafts (green line is also shown in Figure 1E). (**D**) H&E-stained sections from *S.epi*-monocolonized syngeneic grafts in hosts with anti-*S.epi* memory or a syngeneic, uncolonized graft in a naive host. A blinded pathologist identified (black arrows) and quantified (purple bars) abnormal mixed lymphocytic/neutrophilic infiltrates around hair follicles. Quantifications are plotted with the macroscopic 4-point score of the graft at sacrifice (orange bars). Original magnification ×20. (**E**) PCR band intensities of *S.epi* DNA isolated from skin swabs of *S.epi*-painted SPF hosts naive to (green) or with memory against (orange) *S.epi*. Intensities are normalized to a *S.epi*-positive control. An unpaired, 2-tailed *t* test was performed at each time point. (**F**) Graft scores of syngeneic hosts with or without anti–*S*. *aureus* memory that received skin grafts from SPF donors with or without *S*. *aureus* colonization. (**C**, **E**, and **F**) The area under the graft score curves was calculated for each mouse, and ANOVA with multiple comparisons was performed. Plots show 1 (**F**), 2 (**E**), 3 (**B** and **C**, green and blue lines), or 4 (**C**, orange line) independent experiments. **P < 0.005, ***P < 0.0005; mem., memory; col., colonized; d, day; ND, no data.

In contrast to laboratory mice in controlled animal facilities, barrier tissues in humans and wild mice have many commensal-specific memory T cells, reflecting exposure to a variety of microbes and environmental antigens over time (4, 6, 15, 16). We therefore asked whether hosts with memory against *S.epi* would damage syngeneic, *S.epi*-colonized skin grafts more than hosts naive to *S.epi*. Because *S.epi* is not a native mouse commensal, we generated anticommensal memory using s.c. immunizations with *S.epi*, as this approach is known to induce local immune responses in the skin (17, 18). SPF mice were immunized s.c. with *S.epi* or not, 1 month before receiving a *S.epi*-monocolonized or GF syngeneic skin graft (Figure 2A). For all 3 transplantation conditions, we tested syngeneic grafts from male donors into male hosts and from female donors into female hosts. In a group of mice without transplants sacrificed 1 month after s.c. *S.epi* immunization, we confirmed that s.c. immunization expanded endogenous *S.epi*-reactive CD8^+^ CD44^+^ T cells in the SDLNs and skin, using a tetramer of the nonclassical MHC H2-M3 presenting a formylated peptide of *S.epi* to identify *S.epi*-reactive CD8^+^ T cells (Figure 2B).

Strikingly, mice with anti-*S.epi* memory damaged *S.epi*-colonized syngeneic skin grafts significantly more than hosts lacking anti-*S.epi* memory (Figure 2C and Supplemental Figure 6A). Because the extent of damage was equal in male and female hosts of syngeneic grafts under all transplantation conditions, the data were aggregated for male and female mice in the same experimental groups. The damage was not due to infection caused by the *S.epi* immunization protocol, since GF skin grafts in hosts harboring anti-*S.epi* memory did not develop any graft damage (Figure 2C and Supplemental Figure 6A). Thus, the damage to *S.epi*-colonized skin grafts observed in SPF hosts with memory to *S.epi* was extensive enough to occur in a syngeneic setting, thus independently from an alloresponse.

Microscopically, colonized grafts in hosts with anti-*S.epi* memory had abnormal lymphocytic and neutrophilic infiltration around hair follicles (Figure 2D). These abnormalities were localized to the graft and not found in host flank skin immediately adjacent to the grafts (Supplemental Figure 6B). *S.epi* is known to associate with keratinocytes at the base of hair follicles (19). Therefore, the graft pathology was consistent with a host immune response against graft-resident *S.epi*.

Notably, over time, the syngeneic colonized grafts in hosts with anti-*S.epi* memory recovered (Figure 2C). This correlated with a resolution of the cellular infiltrates in hair follicles in histological sections (Figure 2D). We hypothesized that this was due to progressive disappearance of *S.epi* colonization, either because the graft-resident *S.epi* was outcompeted by the native mouse microbiota or because it was eliminated by the host’s immune system. To determine the longevity of *S.epi* colonization, mice harboring or lacking anti-*S.epi* memory were painted with *S.epi*. After the last painting, tail skin was swabbed every few days. DNA was isolated from these samples, and putative *S.epi* DNA was amplified by PCR. The presence of anti-*S.epi* memory accelerated *S.epi* elimination from the skin; *S.epi* DNA abundance in tail swabs from mice harboring anti-*S.epi* memory became almost undetectable 18 days after the last painting, while *S*.*epi* DNA from mice without memory was still abundant at that time (Figure 2E). In mice lacking memory, *S.epi* DNA was significantly reduced 32 days after the last painting (Figure 2E). These time frames correlated with how long it took for syngeneic, colonized grafts to start improving (around 20 days after transplantation) in hosts with anticommensal memory (Figure 2C). We hypothesize that graft recovery can occur in syngeneic grafts because there are no alloantigens present to perpetuate the immune response against the transplanted organ. In allograft recipients, however, we propose that this host-versus-commensal immune response would work in parallel to the host-versus-graft immune response to heighten graft damage and accelerate rejection.

To determine whether commensal memory–associated graft damage was unique to *S.epi* or whether it extended to other commensals, we colonized SPF donor mice with a strain of *S*. *aureus* adapted to mouse colonization (20). To improve *S*. *aureus* establishment, the skin of SPF female donor mice was first topically treated with the antiseptic chlorhexidine, and then painted for 5 consecutive days with *S*. *aureus*. Skin colonized with *S*. *aureus* or not was transplanted onto syngeneic hosts that had been immunized or not with *S*. *aureus* a month earlier. A similar pattern was observed to that seen with *S.epi*: some syngeneic skin graft damage occurred in naive hosts that received *S*. *aureus*–colonized syngeneic grafts when compared with grafts without *S*. *aureus*, and this damage was significantly heightened in hosts with preexisting memory to *S*. *aureus* (Figure 2F). Together, these results support the notion that host-versus-commensal immune memory participates in damage to colonized solid organ transplants.

### Anti-commensal memory T cells are required to damage colonized syngeneic skin grafts.

**Figure 3:**
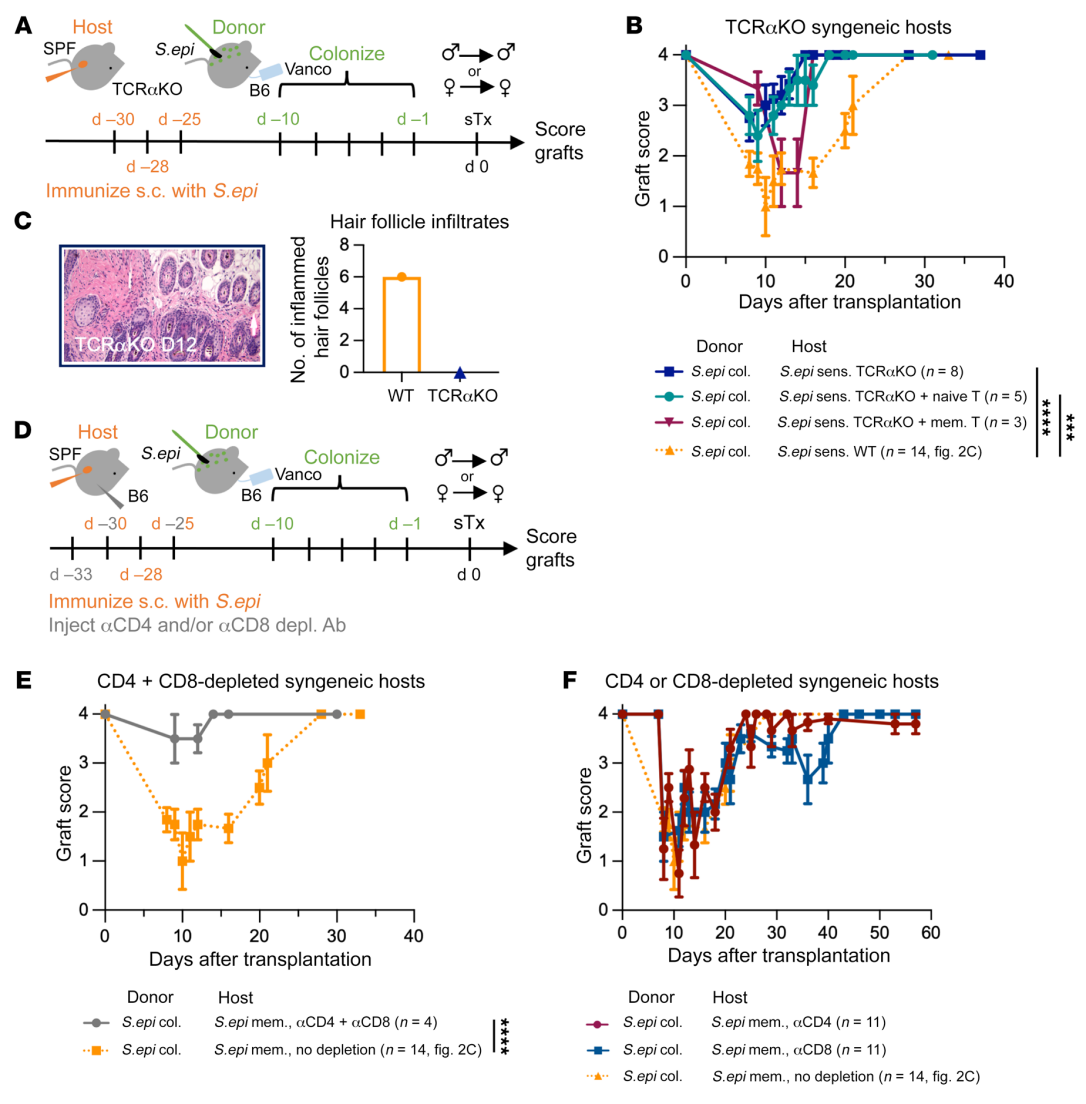
Both CD4^+^ and CD8^+^ host memory T cells are required to damage colonized, syngeneic skin grafts. (**A**) TCRαKO hosts were immunized 3 times s.c. with *S.epi* to induce anti-*S.epi* memory. One month later, they received syngeneic skin grafts from *S.epi*-monocolonized hosts. (**B** and **C**) Graft scores (**B**) and H&E-stained skin graft sections (**C**) from memory-harboring TCRαKO hosts of *S.epi*-monocolonized skin grafts (**B** and **C**, dark blue) compared with curves and images of WT hosts with anti-*S.epi* memory that received *S.epi*-monocolonized, syngeneic skin grafts (**B** and **C**, orange, dotted; same cumulative data shown in Figure 2C). Original magnification ×20. (**B**) Graft scores of TCRαKO mice with anti-*S.epi* memory seeded with T cells from the lymph nodes and spleen of C57BL/6 mice naive to *S.epi* (teal) or T cells from the lymph nodes, spleen, and skin of C57BL/6 mice sensitized s.c. with *S.epi* (maroon) before transplantation with a *S.epi*-monocolonized syngeneic skin graft. Plots show 1 (maroon), 2 (teal), or 3 (dark blue) independent experiments. (**C**) Number of inflamed hair follicles in H&E-stained skin grafts counted by a blinded pathologist. (**D**) Hosts received anti-CD4 (GK1.5) and/or anti-CD8 (2.43.1) i.p. on days –2, 0, and 5 relative to s.c. injection with *S.epi*. CD4^+^ and/or CD8^+^ populations recovered before skin transplantation with a *S.epi*-monocolonized skin graft. (**E**) Anti-CD4 and anti-CD8 codepletion plotted against relevant data from Figure 2C. (**F**) Anti-CD4 (maroon) or anti-CD8 (blue) depletion alone plotted against cumulative data from Figure 2C. Plots combine 3 independent experiments. (**B**, **E**, and **F**) The area under the graft score curves was calculated for each individual mouse and analyzed using ANOVA with multiple comparisons. WT hosts with anti-*S.epi* memory given *S.epi*-colonized skin grafts (dotted orange line) shown in Figure 2C were included in all ANOVA analyses. ***P < 0.0005, ****P < 0.00005; mem., memory; col., colonized; d, day; sens., sensitized.

Given the T cell response to *S.epi* following transplantation (Figure 1, B and C) and the fact that *S.epi* is known to generate a wide array of T cell responses in mouse skin (6, 12), we hypothesized that memory T cells were responsible for the damage observed in hosts with anti-*S.epi* memory. To test this hypothesis, we transplanted *S.epi*-monocolonized skin into syngeneic T cell receptor α–knockout (TCRαKO) hosts immunized a month earlier with *S.epi* (Figure 3A). Compared with the severe graft damage observed in T cell–replete mice, TCRαKO hosts displayed either no damage to their syngeneic skin grafts, or mild damage that resolved quickly (Figure 3B). Further, colonized skin graft sections taken from *S.epi*-sensitized TCRαKO hosts 12 days after transplantation (the peak of damage in WT mice with anticommensal memory) revealed none of the abnormal infiltrates around hair follicles that were present in WT hosts with anti-*S.epi* memory (Figure 3C; controls in Figure 2D). The lack of macro- or microscopic damage to colonized skin grafts in sensitized TCRαKO hosts suggests that T cells are required to damage colonized, syngeneic grafts.

TCRαKO hosts are genetically devoid of αβ T cells, such that they lack both anticommensal memory T cells and all other αβ T cells at transplantation. To ensure that the protective phenotype we observed was from the lack of anti-*S.epi* memory and not the global lack of T cells, we adoptively transferred T cells from naive or *S.epi*-sensitized WT mice into previously *S.epi*-sensitized TCRαKO mice, 1 day before transplantation with a syngeneic *S.epi*-colonized skin graft. Transfer of T cells from *S.epi*-sensitized but not naive WT mice resulted in graft damage (Figure 3B). The damage in memory T cell–seeded TCRαKO mice did not persist as long as the damage in sensitized WT mice, perhaps because of more rapid clearance of graft-resident *S.epi* due to the high number of adoptively transferred memory T cells.

As an alternative approach to TCRαKO hosts, we used antibodies to deplete CD4^+^ and/or CD8^+^ T cells 1 day before immunizing mice for the generation of anti-*S.epi* memory. We depleted CD4^+^ and/or CD8^+^ T cells again before the second and third *S.epi* immunizations and then allowed the CD4^+^ and CD8^+^ T cell populations to recover before skin transplantation (Figure 3D and Supplemental Figure 7A). We reasoned that this approach would allow us to selectively prevent anti-*S.epi* T cell memory without compromising T cell immunity at the time of syngeneic skin transplantation. In keeping with results observed in TCRαKO mice, hosts lacking CD4^+^ and CD8^+^ T cells during *S.epi* immunization minimally damaged subsequent *S.epi*-colonized syngeneic skin grafts (Figure 3E). Conversely, hosts that received only the anti-CD4 or the anti-CD8 depleting antibody throughout the *S.epi* immunization displayed graft damage similar to that seen in their undepleted counterparts (Figure 3F). Together, these results indicate that damage to colonized, syngeneic skin grafts in hosts harboring anticommensal memory is dependent on αβ T cells. Neither CD4^+^ nor CD8^+^ T cell memory was required for graft damage to occur, suggesting that one memory subset is sufficient for, and both subsets can contribute to, the graft damage.

To address the specificity of the memory response, we investigated whether host memory to unrelated commensals could promote damage of *S.epi*-colonized syngeneic grafts. We hypothesized that memory against bacteria that share epitopes with the commensals on the donated organ would induce graft damage whereas memory against less related strains would not. To this end, we generated host memory against either *S.epi* Tü3298, a strain closely related to the NIHLM087 *S.epi* strain used to colonize the donor skin before transplantation; or *E*. *coli* DH5α, of a distant genus. In one experiment, the immunizing bacteria were heat-killed (HK), to determine whether host recognition of bacterial epitopes and pattern-recognition ligands was sufficient, in the absence of bacterial metabolites, to elicit memory responses that could damage the graft. Indeed, hosts with memory against *S.epi* Tü3298 or HK *S.epi* NIHLM087 significantly damaged *S.epi* NIHLM087–colonized grafts. Conversely, hosts with memory against HK *E*. *coli* DH5α did not (Supplemental Figure 8). Thus, damage to a colonized skin graft mediated by host-versus-commensal memory responses does not require that host memory be generated with the exact bacterial strain present on the donor organ.

### Host tissue-resident memory cells are sufficient to damage colonized syngeneic skin grafts.

**Figure 4:**
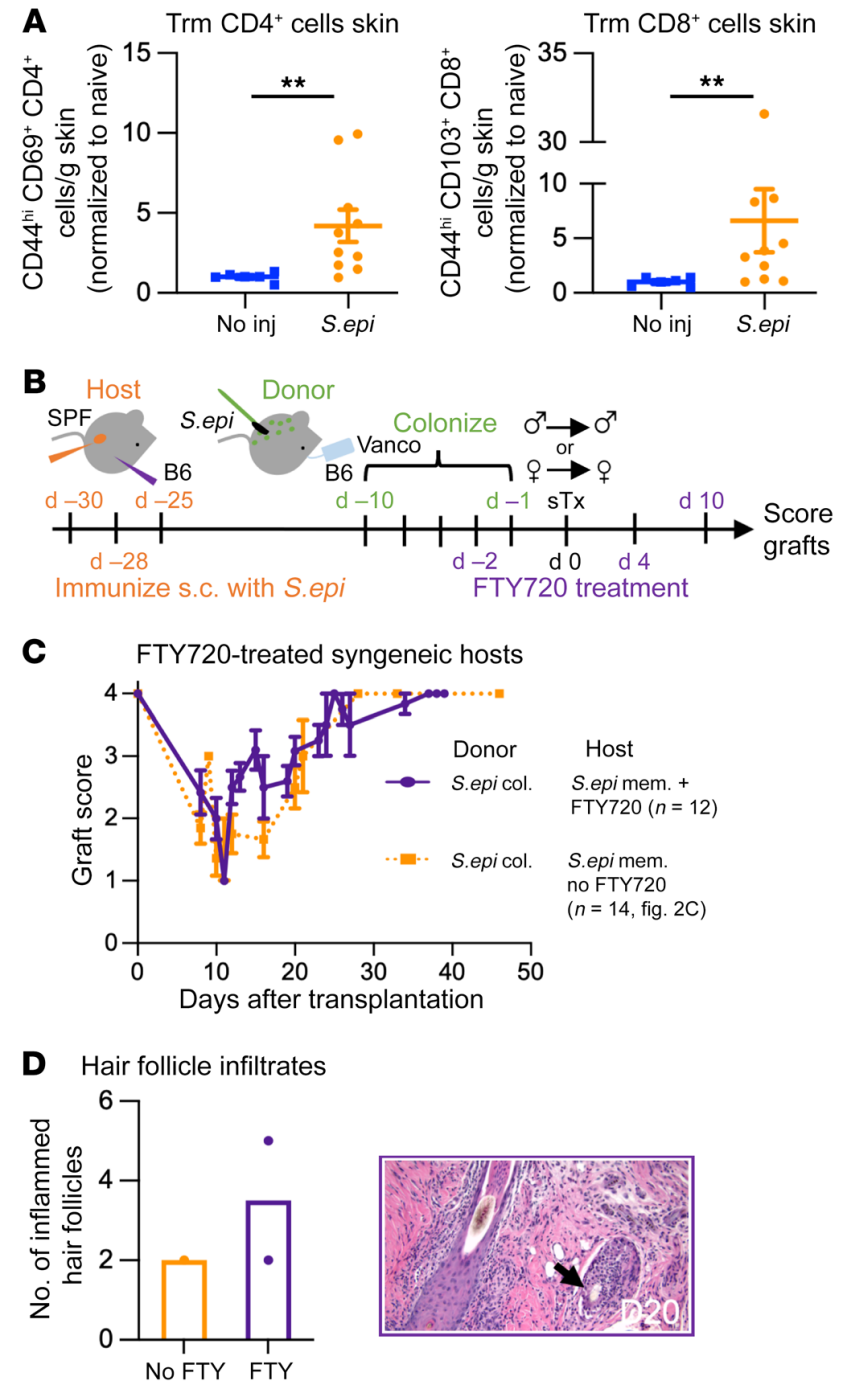
Host tissue-resident memory cells are sufficient to damage colonized, syngeneic skin grafts. (**A**) Trm cells isolated from the skin of mice that were immunized s.c. with *S.epi* one month earlier. Cells gated on CD45.2^+^CD8^–^CD4^+^CD44^+^CD69^+^ (CD4^+^, left) or CD45.2^+^CD8^+^CD4^–^CD44^+^CD103^+^ (CD8^+^, right) populations. Plots show fold change relative to naive mice (mean ± SEM) and include 3 independent experiments analyzed using Wilcoxon’s log-rank test. **P < 0.005. (**B**) Mice harboring anti-*S.epi* memory were injected i.p. with FTY720 on days –2, 0, 4, and 10 relative to transplantation with *S.epi*-monocolonized skin grafts. (**C** and **D**) Graft scores (**C**) and H&E-stained graft sections (**D**) from mice treated with FTY720 (purple line) compared with untreated controls shown in Figure 2C (dotted orange line). Black arrow denotes abnormal immune infiltrates around hair follicles, which were quantified by a blinded pathologist. Original magnification ×20. Part **C** incorporates 3 independent experiments. The area under the graft score curves was calculated for each individual mouse, and ANOVA with multiple comparisons was performed on these values. The curves were not statistically different. mem., memory; col., colonized; d, day.

*S.epi* is known to generate tissue-resident memory T cells (Trm cells) in the skin (8, 12). In our model, the s.c. immunizations that generated anti-*S.epi* memory established a robust population of CD4^+^ and CD8^+^ Trm cells in mouse skin (Figure 4A).

To determine whether Trm cells were sufficient to damage colonized syngeneic skin grafts in hosts with anti-*S.epi* memory, we treated hosts with the sphingosine-1-phosphate receptor (S1PR) agonist FTY720 on days –2, 0, 4, and 10 relative to transplantation of *S.epi*-colonized skin grafts (Figure 4B). FTY720 downregulates S1PR and traps naive and central memory lymphocytes in the lymph nodes, thus revealing the impact of effector memory and Trm cells (21). Two FTY720 injections, with no additional boosters, dramatically reduced the number of circulating T cells for 1 week (Supplemental Figure 9). Preventing recruitment of new naive and central memory T cells into the skin graft using FTY720 did not reduce damage to colonized syngeneic skin grafts compared with damage in untreated hosts (Figure 4C). Skin grafts from FTY720-treated mice had the same hair follicle pathology as those from untreated mice, even 20 days after transplantation, a time point slightly past the peak of graft damage (Figure 4D). These data support the conclusion that Trm and/or effector memory cells are sufficient to potentiate damage against colonized, syngeneic skin grafts.

### Anti-commensal memory resists immunosuppression and accelerates colonized allograft rejection.

**Figure 5:**
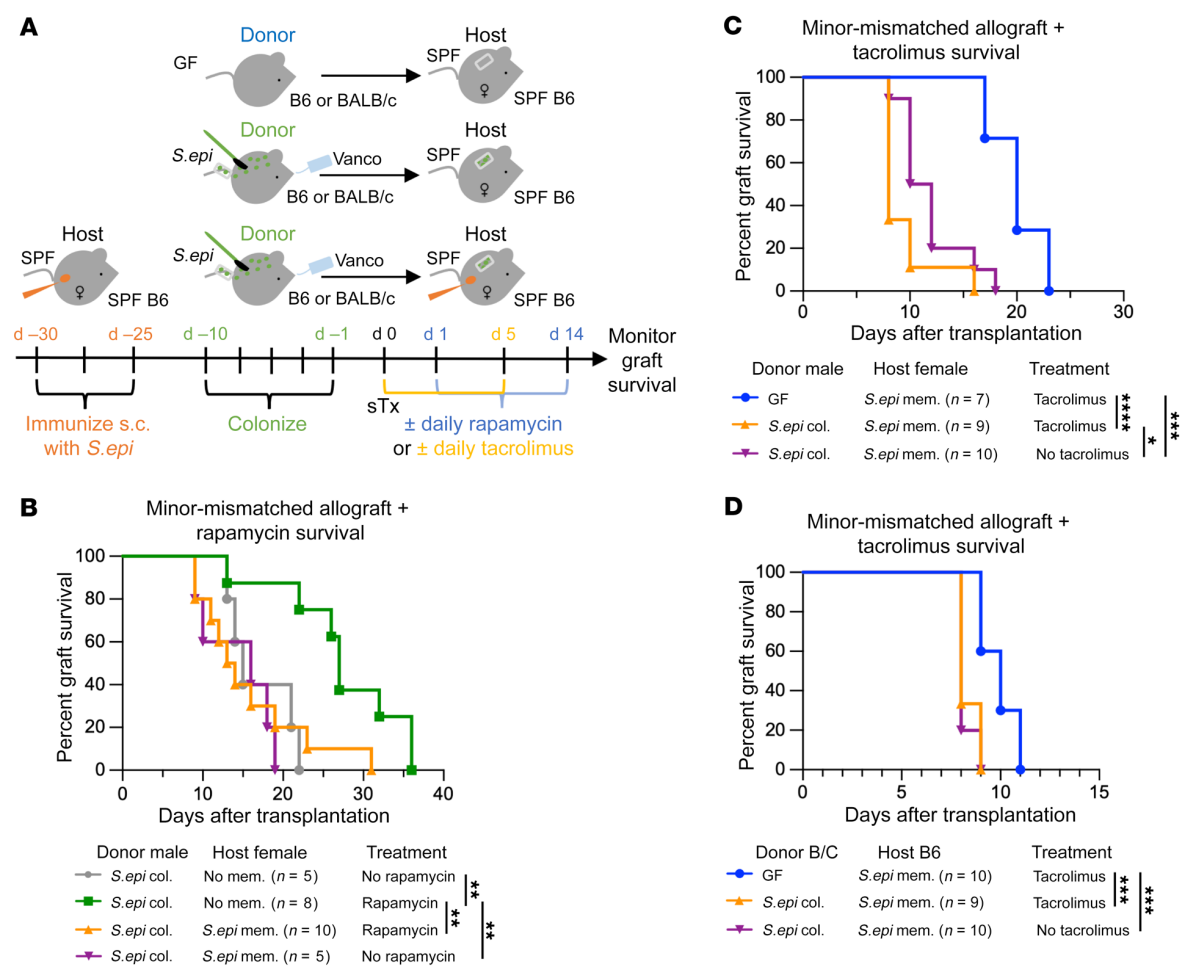
Hosts with memory against graft-resident commensals resist immunosuppression and reject colonized allografts more quickly. (**A**) Female C57BL/6 recipient mice were immunized s.c. or not with *S.epi* to establish anticommensal memory 30 days before transplantation. Hosts received a *S.epi*-monocolonized or GF C57BL/6 male (minor-mismatched) or BALB/c (B/C; major-mismatched) skin graft. A subset of hosts received rapamycin for 14 consecutive days (**B**) or tacrolimus for 6 consecutive days (**C** and **D**) starting the day of transplantation. (**B**–**D**) Allograft survival curves for minor-mismatched (**B** and **C**) or major-mismatched (**D**) skin transplant recipients that received rapamycin (**B**), tacrolimus (**C** and **D**), or no immunosuppression (**B**–**D**, purple and gray lines). All survival curves were analyzed using a log-rank (Mantel-Cox) test. *P < 0.05, **P < 0.005, ***P < 0.0005, ****P < 0.0001; mem., memory; col., colonized.

In the clinic, patients typically receive allogeneic rather than syngeneic organs, and take daily immunosuppression to prevent graft rejection. We therefore asked whether host-versus-commensal immunity would accelerate the rejection of colonized allografts in immunosuppressed hosts. C57BL/6 female hosts received skin grafts from either C57BL/6 male (minor-antigen-mismatched) or BALB/c (major-antigen-mismatched) donors. The donors were *S.epi*-colonized or GF, and the hosts either had or did not have anti-*S.epi* memory. Some groups received rapamycin, an mTOR inhibitor, or tacrolimus, a calcineurin inhibitor, to dampen alloimmunity (Figure 5A). For each immunosuppressant, we used a dose that prolonged the survival of allografts in hosts lacking anti-*S.epi* memory (Figure 5B and Supplemental Figure 10, A and B).

Hosts with memory against *S.epi* rejected *S.epi*-colonized, minor-antigen-mismatched skin grafts faster than hosts naive to *S.epi* (Figure 5B). Strikingly, whereas rapamycin prolonged graft survival in naive hosts, it failed to do so in recipients harboring anti-*S.epi* memory (Figure 5B). Rapamycin also failed to prevent damage of syngeneic skin grafts in hosts with anti-*S.epi* memory (Supplemental Figure 11, A–C), suggesting that rapamycin is unable to control anti-*S.epi* memory.

Rapamycin has been shown to impair wound healing and can promote development of T cell memory (22). Therefore, we tested another immunosuppressive drug, tacrolimus, that is not thought to enhance memory formation and that is routinely used in human patients. As with rapamycin, mice treated with tacrolimus rejected *S.epi*-colonized, minor-mismatched skin grafts faster when they harbored anti-*S.epi* memory (Supplemental Figure 10C). Most importantly, tacrolimus also failed to prolong survival of *S.epi*-colonized skin grafts in hosts with anti-*S.epi* memory; in fact, it slightly accelerated graft rejection (Figure 5C).

Finally, we tested whether host-versus-commensal immunity also impacted graft outcomes in a highly immunogenic, fully MHC-mismatched setting. Tacrolimus as a single agent only modestly prolonged graft survival of BALB/c uncolonized GF skin allografts, whether C57BL/6 hosts harbored anti-*S.epi* memory or not (Figure 5D and Supplemental Figure 10B). Nevertheless, hosts with anti-*S.epi* memory rejected *S.epi*-colonized BALB/c skin grafts significantly faster than uncolonized skin grafts. Further, tacrolimus failed to prolong survival of *S.epi*-colonized BALB/c grafts in hosts harboring memory (Figure 5D). Therefore, we conclude that host-versus-commensal memory immune responses can accelerate rejection of colonized allografts and be a cause of resistance to immunosuppression.

## Discussion

Organs harboring their own tissue-specific microbiome are rejected faster than sterile organs after transplantation (1). Here we show that this accelerated rejection occurs at least in part because of host immune responses against the commensal microbes present on the donated organ. Naive commensal-specific T cells in graft-recipient mice expanded in response to commensals present on transplanted organs and inflicted modest transient damage to the colonized graft. More significantly, memory commensal-specific T cells caused substantial and more durable damage to colonized organs. Trm and/or effector memory T cells in the host were sufficient to drive this damage. This host-versus-commensal immune response was distinct from the alloresponse and resulted in resistance to immunosuppression.

Many prior studies implicate the microbiome in altering transplant outcomes. For example, our group showed that mice pretreated with antibiotics before skin transplantation exhibited delayed rejection compared with untreated mice (23), and that the presence of intestinal communities such as *Alistipes* spp. correlated with longer skin graft survival (24). These studies highlighted the importance of the gut microbiota in skin graft outcomes, but did not address whether the organ-specific microbiome alone was sufficient to impact allograft rejection. To that end, we previously showed that graft *S.epi* can accelerate skin transplant rejection by augmenting the effector phase of the alloresponse in the graft (5). In addition to that mechanism, we show here that anticommensal immune responses contribute to damage of colonized organs. To our knowledge, this is the first study demonstrating that host T cell–mediated immune responses to commensal bacteria on the transplanted organ reduce survival of a colonized transplant. Thus, importantly, our results show that adaptive immune responses beyond the alloresponse can contribute to organ damage.

Our results also demonstrate that host-versus-commensal memory immune responses resist immunosuppression with rapamycin and tacrolimus. This is not entirely surprising because we implicate Trm and/or effector memory T cells in potentiating damage against colonized grafts. Owing to their largely tissue-resident status and their memory phenotype, these cells are harder to suppress with immunosuppressive drugs, which are better at inhibiting newly dividing, naive lymphocytes (25).

In our experiments, colonized syngeneic skin grafts that were damaged by anticommensal memory recovered. We hypothesize that this is because the damaging anticommensal immune response, which localizes around hair follicles, a known *S.epi* niche, successfully eliminates the bacteria and then abates. Given that memory against bacteria closely related to the strain on the skin graft, but not memory against distant bacterial taxa, can perpetuate graft damage, we hypothesize that these immune responses are specific for *S.epi* antigens. However, Gram-negative *E*. *coli* and Gram-positive *S.epi* may train different innate immune programs, and these differences may also account for the findings. Future studies should assess the degree of antigen specificity and characterize (a) which commensal peptides are immunogenic, (b) the pathways through which they signal, and (c) which are the deleterious T cell profiles. Importantly, in this study, the damage we observe cannot be caused by anticommensal memory cross-reacting to alloantigen, because the damage occurs in syngeneic grafts in which no alloantigen is present. It also is not mediated by postsurgical infection caused by *S.epi* colonization on the skin graft, because graft damage is much more severe in colonized graft recipients harboring anticommensal memory than in recipients naive to *S.epi*.

In our model, we establish anti-*S.epi* memory through subcutaneous injections. In human transplant recipients, we hypothesize that Trm cells naturally accumulate against many commensals and can perpetuate chronic damage to colonized organs. In fact, mouse studies show that steady-state microbial colonization in adult life and minor barrier damage that exposes the hosts to commensals in pathogenic contexts establish a cohort of Trm cells poised to attack microbes in colonized organs (11, 13). Indeed, when *S.epi* is painted on adult mouse skin at steady state, it generates Trm CD4^+^ and CD8^+^ cells that preferentially adopt type 17 and type 1 programs (12). Some of these IL-17A–producing CD8^+^ T cells harbor mRNA for the type 2 transcription factor GATA3. They aid in wound repair after tissue injury (11). However, when mice colonized with *S.epi* as adults receive punch wounds or minor skin abrasions and then are repainted with *S.epi*, they mount proinflammatory responses against *S.epi* that seem to outweigh wound-healing benefits (13). Therefore, the type 1 and type 17 cells induced by steady-state colonization do attack *S.epi* in pathogenic contexts. Whether these are the T cell profiles involved in damaging colonized skin grafts remains to be determined.

Additionally, how commensal-specific Trm cells in the host’s skin migrate into colonized skin grafts, or whether only effector memory T cells recruited into the graft mediate damage of colonized skin grafts, needs to be investigated. In the skin, Trm cells localize most strongly to the site where antigen was first encountered and provide the strongest protective immunity in that region (26). However, CD8^+^ Trm cells induced in one part of the skin can confer protective immunity to the same bacterial infection at other skin sites (26). Indeed, a small proportion of CD8^+^ Trm cells migrate away from the initial infection site and randomly disperse throughout the epidermis, where they defend the host at distal locations (27, 28). Additionally, in a cancer setting, activated tumor antigen–specific CD8^+^ Trm cells in the skin caused cross-presenting dermal dendritic cells to mature and migrate to the lymph nodes, where they activated additional T cells that attacked tumor antigen–expressing cells both in, and far from, the initial Trm-rich region (29). CD4^+^ Trm cells are less migratory than CD8^+^ Trm cells. Nonetheless, a proportion of them transiently downregulate CD69, exit the dermis, and recirculate (30). These CD4^+^ ex-tissue-resident memory T cells can activate additional immune responses in the lymph node or reenter the tissue from which they exited at the same or a different site (31, 32). These migratory capacities of both CD4^+^ and CD8^+^ memory T cells indicate that host Trm cells induced upon s.c. *S.epi* immunization may migrate laterally or through circulation into the graft, which is adjacent to the injection site, and damage it. It is possible that lateral migration and/or recirculation could be upregulated in response to the tissue injury conferred by surgery. Exactly how and where graft-derived *S.epi* antigens are presented to memory T cells, and to what extent surgical trauma or reperfusion injury is required to initiate the memory-dependent damage, remain to be determined.

In our experiments, we observe minimal and early damage to *S.epi*-colonized grafts even in immunized hosts that lack T cells. This damage could be mediated by T cell–independent antibody responses or, given its rapid onset and recovery, driven by trained innate memory. Invertebrates that lack adaptive immunity develop allospecific responses (33–35). Similar innate mechanisms exist in vertebrates and contribute to organ rejection. Rag^–/–^ mice that lack B and T cells can reject allografts when they have been preimmunized with donor antigens (36, 37). Further, for at least 1 month after sensitization, macrophages retain “trained” responses that are sufficient to reject allografts in an antigen-specific manner (38). Therefore, trained innate memory against microbial antigens that accompany transplanted organs might also contribute to colonized organ rejection (39). The mechanisms through which macrophages transmit this memory beyond their possibly short lifespan, and whether trained innate memory is relevant for transplant outcomes if established more than 1 month before transplantation, remain to be fully determined.

Our results suggest that graft sterilization prior to transplantation might result in better outcomes for barrier organ allografts. Perfusing the organs with antibiotics shortly before transplantation may reduce donor commensal load and enable colonization of the graft by host commensals. However, antibiotics may also eliminate pro-tolerogenic donor commensals, as some bacteria are known to promote Treg expansion (40). We conclude that memory T cells specific for select bacterial commensals can significantly damage colonized skin grafts independently from an alloresponse and render hosts more resistant to immunosuppression.

## Methods

### Mouse strains and protocols

C57BL/6 and BALB/c mice aged 6–8 weeks were ordered from the same room at Harlan Envigo to control for their vendor microbiota. TCRαKO mice (B6.129S2-Tcra<tm1Mom>/J, stock 002116) were ordered from The Jackson Laboratory and bred in our mouse facility. Bowie mice containing CD8^+^ TCR-transgenic T cells specific for a formylated peptide of *S.epi* (11) were sent to the University of Chicago from the Belkaid laboratory at the NIH. OT I mice containing CD8^+^ TCR-transgenic T cells specific for ovalbumin (41) on a Rag^–/–^ background were bred at the University of Chicago. C57BL/6 and BALB/c gnotobiotic mice were derived by the University of Chicago’s gnotobiotic core facility.

### Bacterial strains and culture

The strains used in this study were *Staphylococcus epidermidis* NIHLM087, *Staphylococcus epidermidis* Tü3298, *Staphylococcus aureus* WU1, and *Escherichia coli* DH5α. Glycerol stocks for all strains were kept at –80°C. For culture, stocks were briefly removed from the freezer, poked with a pipette tip, and immediately returned to –80°C. For NIHLM087 and WU1, the pipette tip was deposited into 5 mL sterile Tryptic Soy Broth (TSB; BD Biosciences) and cultured for 24 hours in a 1*g*, 37°C shake incubator alongside a negative control. Tü3298 was incubated for 48 hours at 37°C in TSB. DH5α was incubated at 1*g* at 37°C for 18–24 hours in 5 mL lysogeny broth (LB).

### S.epi painting

#### Germ-free mice.

NIHLM087 cultures grown as described above were removed from the incubator and immediately delivered to the gnotobiotic facility in a clean, sealable plastic bag. Members of the gnotobiotic facility thoroughly sterilized the tube and introduced it into the flexible firm isolator alongside sterile cotton swabs. Swabs were dipped into the tube carrying *S.epi* and rubbed all over the flank, tail, and ears of the mice. Each mouse was colonized with about 10^8^–10^9^ CFU every 2 or 3 days for a total of 5 applications over 10 days. For their entire time in the isolator, mice received 500 mg/L vancomycin (Hospira) in their drinking water to kill *S.epi* that entered their intestine from grooming. Skin-restricted colonization was verified by placing of skin swabs and fecal samples from colonized mice in 5 mL TSB buffer and incubation of the samples for 24–48 hours in the 1*g*, 37°C shake incubator. All samples were taken at least 1 day after a painting event to ensure measured bacteria were stably incorporated on the skin and not recently applied. Mice with cloudy (bacteria-containing) skin sample cultures and clear fecal sample cultures were considered skin-monocolonized.

#### SPF mice.

Chlorhexidine (Mölnlycke Health Care) was painted onto the tails of SPF mice for 5 consecutive days. Then NIHLM087 cultures grown as described previously were centrifuged at 657 *g* for 5 minutes and resuspended in 1 mL sterile 1× PBS. About 10^8^–10^9^ CFU of *S.epi* were painted on the flank, tail, and ears of conventional (commensal-carrying) mice using sterile cotton swabs. Mice were painted 5 times over the course of 1 week.

### S.epi-specific PCR

Tissues painted with *S.epi* were swabbed with a sterile Puritan swab, placed in enzymatic lysis buffer on ice, and frozen at –80°C until DNA extraction. DNA was extracted using reagents from the Master Pure Yeast DNA Purification Kit and the Qiagen QIAmp Fast DNA Stool Mini Kit. *S.epi* DNA was amplified using forward primer 5′-TTTATCGGAGGTCCAAGCGAA-3′ and reverse primer 5′-ACGGGCAAAAACACTGTCAT-3′ and run on a 1.5% agarose gel. Gels were analyzed using ImageJ software (NIH).

### Recipient immunizations

NIHLM087, Tü3298, WU1, or DH5α cultures grown and centrifuged as described above were resuspended in 1 mL sterile 1× PBS (NIHLM087, WU1, DH5α) or 600 μL 1× PBS (Tü3298). NIHLM087, WU1, or DH5α cultures were diluted 1:5 in sterile 1× PBS in Eppendorf tubes, mixed thoroughly, and kept on ice. Resuspended solutions of Tü3298 were not diluted further. For experiments with heat-killed bacteria, diluted suspensions were incubated at 95°C for 1 hour. For all strains, 1 × 10^5^ CFU (150 μL) of the bacterial suspensions were injected s.c. into SPF mice above the right hind leg using insulin syringes. Recipients were immunized 2 or 3 times over the course of 1 week and left for 1 month to develop immune memory.

### Depleting antibody injections

Mice were bled to quantify CD4^+^ and CD8^+^ T cell populations before treatment. Then they were injected i.p. with 10 mg/kg (250 μg/mouse) of anti-CD4 (clone GK1.5, Bio X Cell) or anti-CD8 (clone 2.43.1, Bio X Cell) antibody diluted in 200 μL PBS per mouse 3 days before the first *S.epi* immunization. One day later, they were bled, and their circulating lymphocytes were analyzed by flow cytometry alongside the pre-injection blood samples to ensure that the antibodies effectively depleted the targeted cell subset. Mice were injected i.p. with the same concentration of anti-CD4 or anti-CD8 again on the first and third days of s.c. *S.epi* immunization. Mice were left for 6 weeks to recover CD4^+^ and CD8^+^ T cell populations before transplantation. This recovery was verified using flow cytometry on longitudinal blood samples.

### FTY720 injections

FTY720 (Enzo Life Sciences) was resuspended in 100% ethanol and stored in aliquoted stock solutions at –20°C. For injection, stock solutions were diluted in filter-sterilized distilled water and not PBS to improve the drug’s solubility. Each recipient mouse was injected i.p. with 20 μg FTY720 in a 60 μL injection volume and monitored carefully to ensure that it did not suffer from alcohol poisoning or osmotic imbalance. Injections were performed 3 days and 1 day before transplantation, and injection success was measured by the absence of circulating lymphocytes in recipient blood analyzed by flow cytometry. Four and ten days after transplantation, mice were again injected i.p. with the same dose of FTY720.

### Transplantation

One-square-centimeter skin grafts taken from the tail of donor mice were transplanted onto the recipient’s flank. Donor mice were anesthetized with ketamine (65–80 mg/kg) and xylazine (5–8 mg/kg) and then euthanized by cervical dislocation. Tails were removed from the mouse body at the base, a midventral incision was made along the tail, and skin was peeled away from the underlying tissue. Skin was cut into 5 skin grafts and rested on ice while the recipients were prepared. Recipients received preemptive analgesia before being anesthetized with ketamine and xylazine. Their flanks were shaved using an electric razor. A portion of flank skin was cut away and replaced with the prepared graft. Recipients were bandaged and monitored for 48 hours after surgery to ensure that they were healthy and active. Bandages were removed 7 days after transplantation. Grafts were checked every other day.

### Graft scoring

Grafts were checked every other day or every 3 days after bandage removal and scored on a 4-point scale and/or monitored for rejection. Grafts received 1 point each for presence of hair, presence of pigment, lack of red spots, and a large graft size. To ensure consistent scoring and minimize the potential for scorer bias, zero points were awarded only when no hair, no pigment, at least one spot, or less than 25% of the initial graft size was observed. Allografts were considered rejected when the grafts had shrunken to less than 25% of the original size and lost all pigment and hair.

### Immunosuppression

Rapamycin powder (Alfa Aesar) was resuspended to 50 mg/mL in 100% ethanol. The stock was diluted to 30 mg/kg in a 5% dextrose solution. Mice were gavaged with rapamycin every day for 14 days after transplantation.

Tacrolimus (FK506, Astellas) was purchased at a concentration of 5 mg/mL and diluted in sterile saline for i.p. injection of 0.1 mg/kg (minor-mismatched grafts) or 1.0 mg/kg (major-mismatched grafts) every day for 6 days, starting on the day of transplantation.

### Leukocyte isolation

#### Skin.

Approximately 2-by-3-cm portions of the flank were shaved before collection of the whole flank (2 × 3 cm) or skin grafts on the back (1 × 1 cm). All specimens were cut into small pieces and placed into a 50 mL Falcon tube on ice carrying 5 mL (whole flank) or 2.5 mL (graft) digestion buffer: 1:10 DNase, 1:50 Liberase (Roche), and cRPMI to volume. cRPMI was made by addition of 2 μL β-mercaptoethanol and 5 mL HEPES solution to 500 mL RPMI stocks. Media tubes were weighed before and after specimens were added to calculate the mass of the harvested skin. Skin was digested for 2 hours in a 37°C shake incubator at 1*g*. The digested product was filtered through a 5 μm cell strainer into a Petri dish, and the tissue was smashed with the rubber end of a 1 mL plastic syringe plunger. Digestion tubes were rinsed with 5 mL RPMI, and the rinse solution was filtered through the cell strainer. The strained product was transferred to a 15 mL Falcon tube and centrifuged for 5 minutes at 370 *g*. The supernatant was removed, and cells were resuspended in cRPMI for counting.

#### Lymph nodes.

Brachial, axial, and inguinal lymph nodes (skin graft–draining lymph nodes [SDLNs]) or all SDLNs plus cervical and mesenteric lymph nodes were dissected out and placed into 5 mL complete DMEM (cDMEM; 500 mL cDMEM contained 5 mL HEPES, 5 mL nonessential amino acids, 2 μL β-mercaptoethanol, 5 mL penicillin/streptomycin antibiotic, and 5% or 10% FBS). Samples were smashed with the rough edge of coated microscope slides and then filtered through 5 μm mesh into a 15 mL Falcon tube. Cells were centrifuged at 370*g* for 5 minutes. The supernatant was removed, and cells were resuspended in cDMEM for counting.

#### Spleen.

Spleens were harvested from mice and placed into 5 ml cDMEM. Spleens were transferred into cell strainers in Petri dishes, where they were smashed with the plastic end of a 1 mL syringe plunger into 2 mL ACK lysis buffer. Once red blood cell lysis was complete (~5 minutes), 8 mL cDMEM was added to the Petri dishes, and the strained cells were transferred to a 15 mL Falcon tube, centrifuged as described previously, and prepared for counting.

### CFSE labeling

Up to 50 × 10^6^ lymphocytes were resuspended in 1 mL 1× PBS plus 5% FBS. The same volume of 1× PBS was prepared in a separate tube and mixed with Cell Trace CFSE dye (Thermo Fisher Scientific) diluted 2:1000 in the volume. The CFSE-containing mixture was added slowly to the cell mixture while vortexing to ensure that single cells were evenly exposed to the dye. The mixture was covered with tin foil to block light and incubated for 5 minutes at room temperature. The reaction was quenched with 3 volumes of cDMEM containing 10% FBS. Stained cells were spun down for 5 minutes at 370*g* and resuspended in 1× PBS. Before incubation in vitro or injection into mice, cells were counted on an Accuri C6 cytometer (BD Biosciences) and were checked for CFSE-positive fluorescence on this same machine.

### Cell injections

Cells were counted and resuspended in sterile 1× PBS to the desired cellular concentration for injection. Cells were injected i.p., retro-orbitally, or s.c. into mice in the SPF facility.

### Histology

Skin grafts were harvested from recipient mouse flanks. They were trimmed to include minimal tissue on the anterior and posterior edges of the graft and some excess recipient flank skin on the lateral edges. Grafts were cut in half transversely, placed in a tissue cassette, and immediately placed in 15–20 tissue volumes of 10% neutral-buffered formalin for 36–48 hours. Cassettes were rinsed once in 70% ethanol and then kept in 70% ethanol until embedding.

All embedding, sectioning, and staining were performed by the University of Chicago Human Tissue Resource Center. Samples were embedded on-edge, cut-side-down in paraffin. Sections were taken from the cut side, ensuring that analyzed tissue was from the center of the grafts. Slides were stained with H&E and given to a blinded pathologist for analysis.

### Antibody staining protocols

#### Live/dead staining.

Counted cells were vortexed, and the desired number of cells for analyses was removed from these bulk mixtures. Cells were placed in FACS tubes and centrifuged at 370*g* for 5 minutes. Supernatants were removed, and cells were resuspended in 200 μL 1× PBS containing 1:1000 Live/Dead Aqua stain by volume (Life Technologies). Cells were incubated for 20 minutes at room temperature in the dark.

#### MHC-I tetramer staining.

Cells stained with live/dead dye were washed and spun down at 370*g* for 5 minutes. Supernatants were flicked off, and cells were resuspended in 20 μL FACS buffer per 10^6^ cells (in many cases, the residual volume in the tubes was used and no additional volume was added). To this volume, an appropriate amount of tetramer (1:200 dilution by volume for H2-M3:fMIIINA) and Fc block (1:500 by volume) was added. Cells were stained at room temperature in the dark for 20 minutes.

#### Surface antibody staining.

Cells stained with live/dead dye and, in some cases, tetramer were washed and centrifuged at 370*g* for 5 minutes. Supernatants were removed, and cells were resuspended in 50 μL of a FACS solution containing 1:200 dilutions of all surface antibodies desired for the experiment. These included CD45.2-APC (104, eBioscience), CD45.2-APCCy7 (104, eBioscience), CD45.1-PECy7 (A20, eBioscience), CD45.1-eF450 (A20, eBioscience), CD45.1-APCCy7 (A20, eBioscience), CD4-BV605 (RM4-5, BioLegend), CD8-BV711 (53-6.7, BioLegend), CD103-FITC (2E7, eBioscience), CD69-BV421 (FN50, BioLegend), TCRβ-APCe780 (H57-597, eBioscience), CD44-PECy7 (IM7, eBioscience), CD44-eF450 (IM7, eBioscience), and B220-V500 (RA3-6B2, BD Horizon). Mixed samples were incubated for 15 minutes in the dark at room temperature. Single-stain controls used to calibrate the flow cytometer were prepared for each fluorophore used in the experiment.

### Flow cytometry

Stained samples were run on the LSR Fortessa 4-15 HTS (BD Biosciences), the LSR Fortessa 4-15 (BD Biosciences), or the Accuri C6 Flow Cytometer (BD Biosciences) in the University of Chicago Cytometry and Antibody Technology Core Facility. Data were collected by FACSDiva software.

### Statistics

Flow cytometry data were analyzed by FlowJo (BD Biosciences). Quantified data were processed in Microsoft Excel, and statistical analyses were performed in GraphPad Prism. The threshold for significance in all cases was *P* < 0.05. Statistical tests used include the unpaired, 2-tailed *t* test, Welch’s unpaired, 2-tailed *t* test, Wilcoxon’s signed-rank test, area under the curve, ANOVA followed by Tukey’s multiple-comparison test, and log-rank (Mantel-Cox) survival analysis.

### Study approval

All animal experiments were approved by the University of Chicago’s Institutional Animal Care and Use Committee. The University of Chicago’s animal care and use program is accredited by the Association for Assessment and Accreditation of Laboratory Animal Care International, and animals were maintained in accordance with the *Guide for the Care and Use of Laboratory Animals*, 8th edition (National Academies Press, 2011).

## Author contributions

IP, MS, YML, and MLA developed the study. LC, YW, and BT conducted surgeries and developed surgical techniques. IP, MS, ZL, and RL performed experiments. YB shared bacterial strains, protocols, and experimental insights. HS analyzed histological sections. ASC provided feedback on the study design. IP and MLA wrote the manuscript, and all authors edited it. First-authorship order reflects experimental and writing contributions.

## Supplementary Material

Supplemental data

## Version 1 07/14/2022

In-Press Preview

## Version 2 09/01/2022

Electronic publication
