# Supplementary material for: Host-versus-commensal immune responses participate in the rejection of colonized solid organ transplants
Source: J Clin Invest. 2022 Sep 1;132(17):e153403. doi: 10.1172/JCI153403 (PMC9435649; doi:10.1172/JCI153403)
Supplement: Supplemental data [file jci-132-153403-s053.pdf]

Supplementary Figure Legends

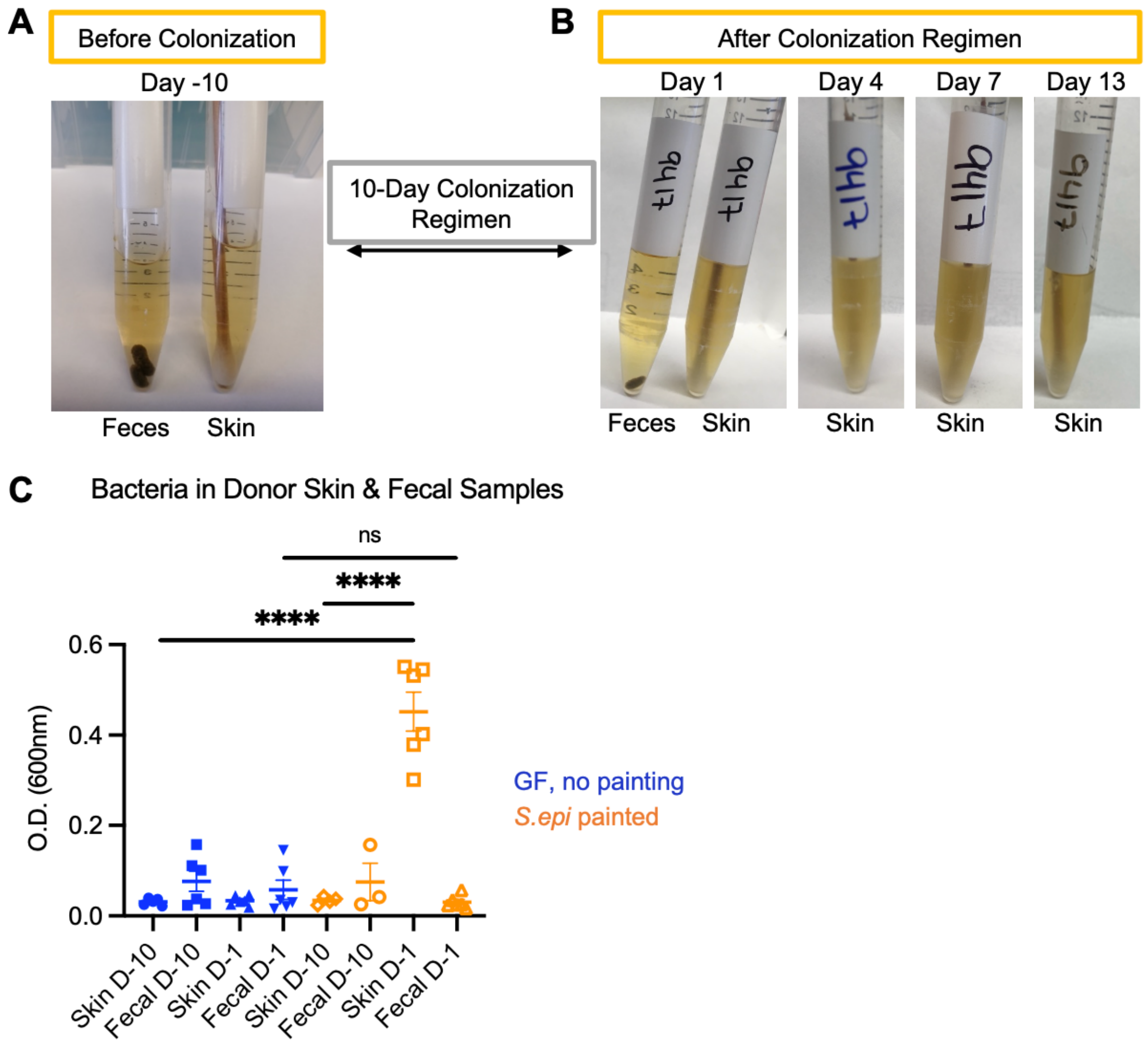

Supplementary Figure 1: The protocol for *S.epi* painting of GF mice induces selective colonization of mouse skin that is stable for at least two weeks. (A) Skin swab and fecal sample cultures from a skin graft donor before colonization. (B) Fecal sample (day 1, left) and skin swabs (days 1, 4, 7, and 13) from mice painted following the protocol outlined in Figure 1A. Cloudy media indicates bacterial growth, clear media indicates a sterile sample. Day 0 is the last day of the 10-day colonization regimen. No additional painting occurred after that time. (C) Quantified bacterial growth in skin and fecal swabs from donor mice painted (*S.epi*) or not (GF) with *S.epi* following the protocol described in Figure 1A. Swabs and fecal pellets were cultured for 24h prior to measurement. D-10: samples were taken before painting the mice on this day. D-1: second-to-last day of colonization protocol.

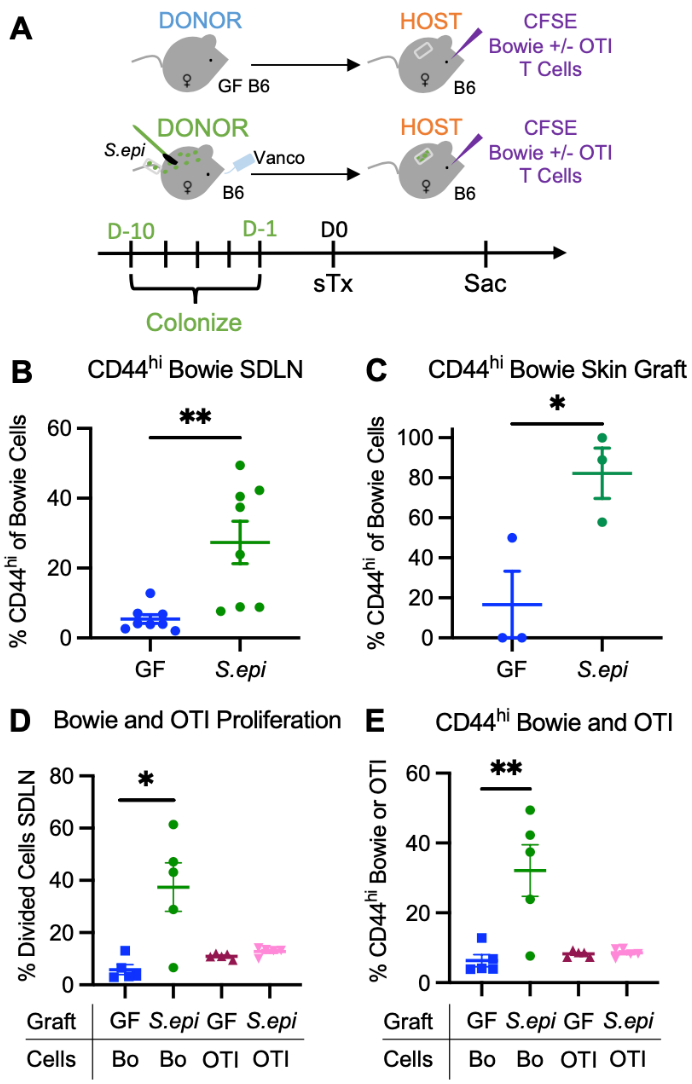

Supplementary Figure 2: Hosts mount a *S.epi*-specific T cell response to *S.epi*-colonized syngeneic skin grafts. (A) Skin graft recipients were seeded with CFSE-labeled *S.epi*-specific Bowie T cells ($1x10^6$ cells) (B-C) or Bowie (Bo in legend) T cells and OVA-specific OTI (OTI in legend) T cells ($1x10^6$ of each) (D-E). Hosts then received a GF or *S.epi*-mono-colonized syngeneic skin graft. $CD44^{hi}$ Bowie and/or OTI cells were examined 6 (B, D-E) and 10 (C) days post-transplantation in the SDLN (B, D-E) and skin graft (C). Plots represent Mean+/-SEM and were analyzed using Welch's unpaired t test. Results were pooled from three (B) independent experiments. *$p<0.05$, **$p<0.005$.

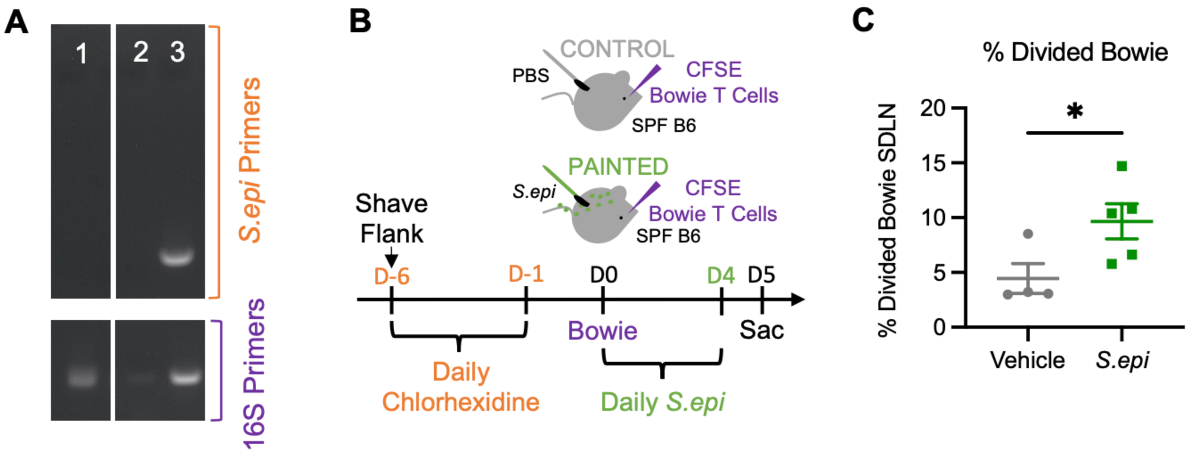

Supplementary Figure 3: *S.epi* does not colonize skin of SPF mice housed in our mouse facility. (A) *S.epi*-specific (top row) and 16S (bottom row) PCR of DNA isolated from swabbing the skin of a mouse housed under SPF conditions (lane 1), from the materials and buffer used for isolation (lane 2), or from the tail swab of a *S.epi*-mono-colonized gnotobiotic positive control mouse (lane 3). (B) SPF mice were shaved, painted with chlorhexidine for 5 consecutive days to open a niche for *S.epi*, and then seeded with $1x10^6$ CFSE-labeled Bowie T cells. One group then was painted with PBS (vehicle control) and the other with *S.epi* for 5 consecutive days. All mice were sacrificed 1 day after the last painting. (C) Bowie T cell proliferation in mice painted or not with *S.epi*. Data were analyzed using an unpaired t test, $*p<0.05$.

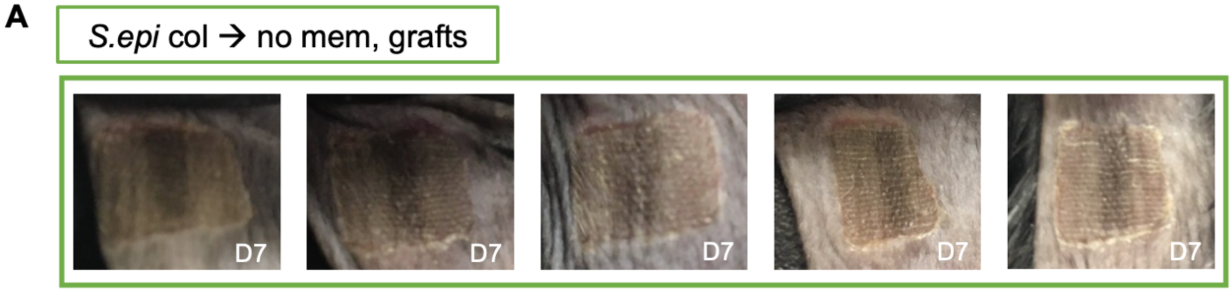

Supplementary Figure 4: *S.epi*-colonized skin grafts show no signs of infection at bandage removal. (A) Photographs of seven-day-old *S.epi*-mono-colonized skin grafts, taken at bandage removal. Grafts show no signs of infection or damage. Grafts are large with full pigment, full hair, and no red spots, or purulence.

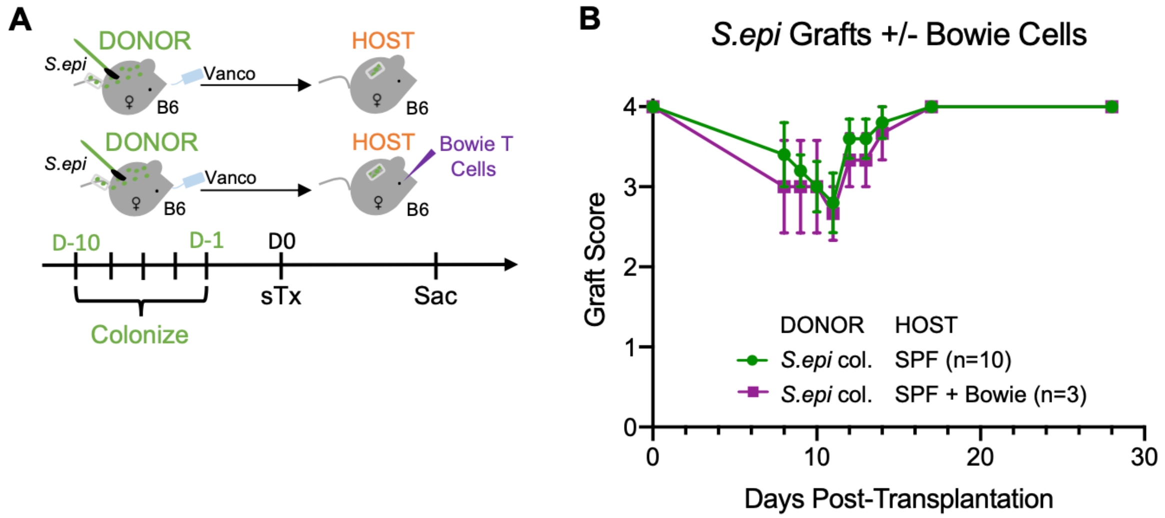

Supplementary Figure 5: Addition of naïve CD8^{+} graft-commensal-reactive T cells does not perpetuate major damage to colonized, syngeneic skin grafts. (A) *S.epi*-mono-colonized skin graft recipients that were seeded (purple line) or not seeded (green line) with 1.5x10^{6} enriched Bowie T cells one day before transplantation. (B) Four-point graft scores over time analyzed as in Figure 2C. Curves are not significantly different.

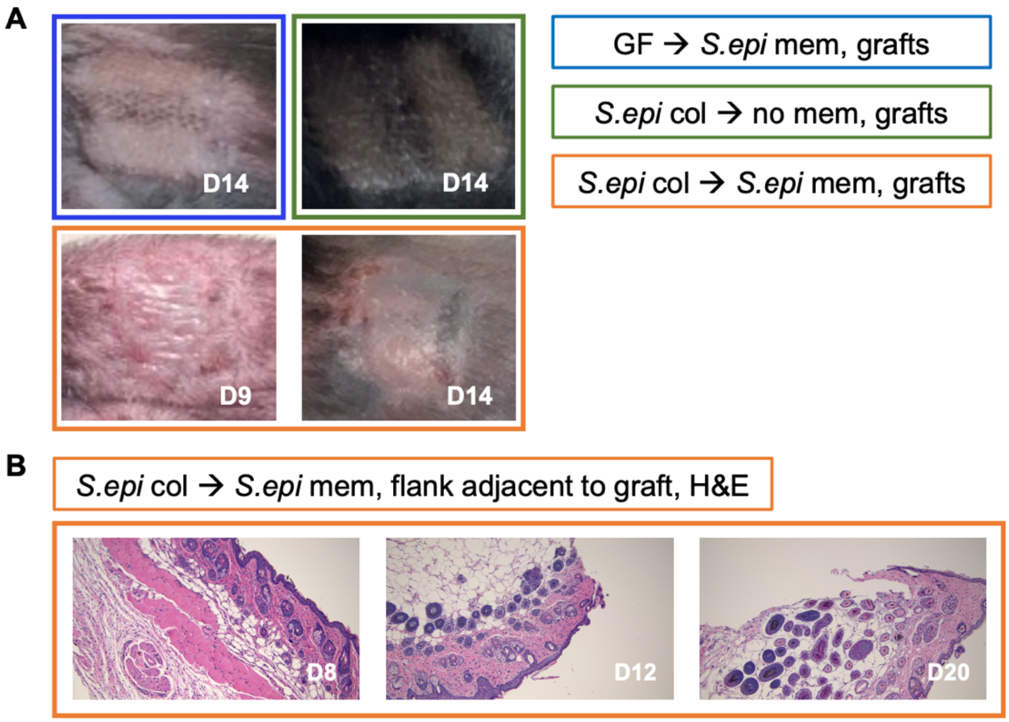

Supplementary Figure 6: Hosts with anti-*S.epi* memory damage syngeneic, *S.epi*-mono-colonized skin grafts in a manner localized to the graft and not caused by infection. (A) Images of skin grafts from SPF syngeneic hosts without (green box) or with (blue and orange boxes) anti-*S.epi* memory that received GF (blue box) or *S.epi*-mono-colonized (green and orange boxes) skin grafts. (B) Images of H&E-stained histological sections taken from host flank skin immediately adjacent to skin grafts shown in Figure 2D. Grafts are *S.epi*-mono-colonized syngeneic transplants in hosts harboring anti-*S.epi* memory. A pathologist blinded to the experimental groups found no abnormalities in any of these graft-adjacent flanks.

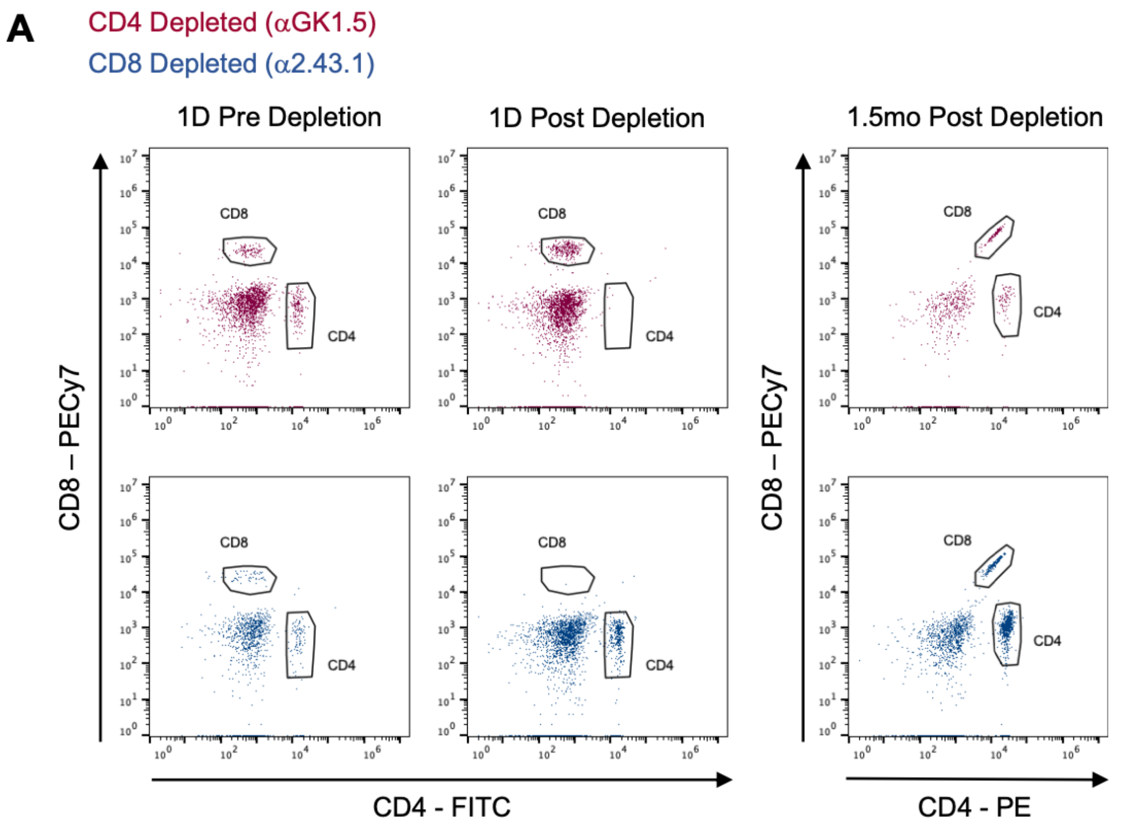

Supplementary Figure 7: Impact of anti-CD4^{+} or anti-CD8^{+} treatment on circulating T cells. (A) Flow cytometry on blood samples from mice injected intraperitoneally with anti-CD4 (clone GK1.5, maroon) or anti-CD8 (clone 2.43.1, blue) mAbs drawn one day before, one day after, and 1.5 months after injection. Gated on bulk lymphocytes (one day pre-injection, one day post-injection) or on CD45.2^{+} lymphocytes (1.5 months post-injection).

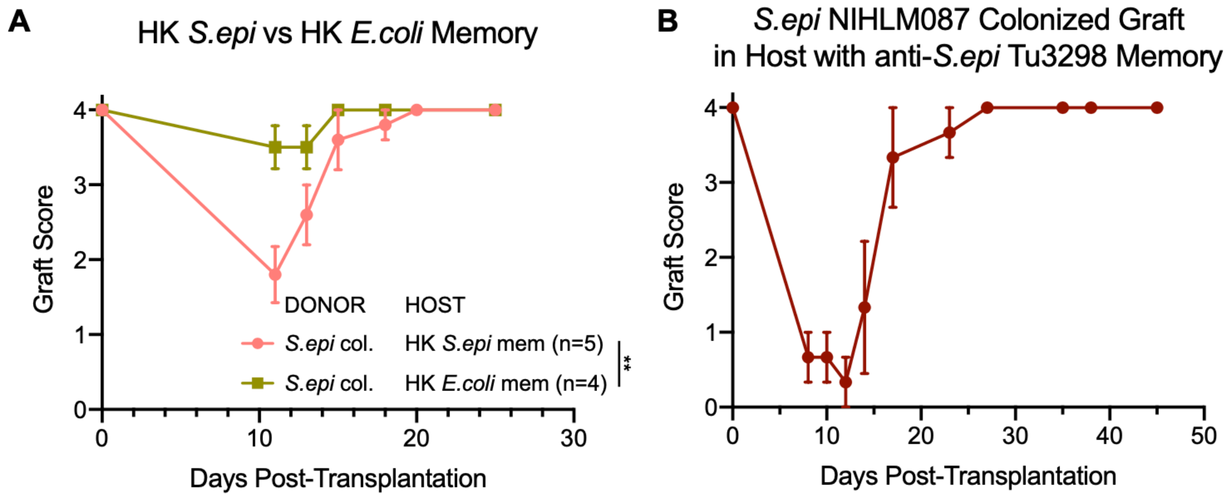

Supplementary Figure 8: Memory against species-matched commensals on donated organs, but not against genus-mismatched commensals, potentiates colonized graft damage. (A) Mice were subcutaneously immunized with heat-killed *S.epi* NIHLM087 or heat-killed *E.coli* DH5α one month before transplantation with a syngeneic skin graft mono-colonized with live *S.epi* NIHLM087. Grafts were scored on a four-point scale. (B) Mice were subcutaneously immunized with *S.epi* Tü3298 one month before receiving a skin graft mono-colonized with *S.epi* NIHLM087. Grafts were scored on a four-point scale. The area under the graft score curves was calculated for each individual mouse and an ANOVA analysis with multiple comparisons was performed on these values. n=3. **$p<0.005$.

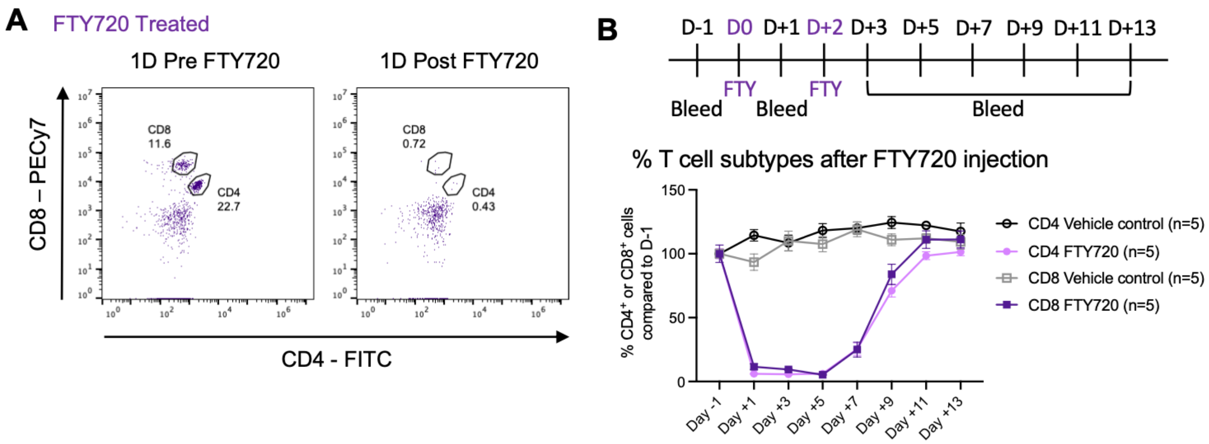

Supplementary Figure 9: Impact of FTY720 treatment on circulating T cells. (A) Flow cytometry on blood samples from mice injected intraperitoneally with FTY720 taken one day before or one day after injection. Gated on bulk lymphocytes. All data were collected on an Accuri C6 Cytometer. (B) Circulating $CD4^+$ or $CD8^+$ lymphocytes in mice injected with 2 doses of 20μg FTY720 on day 0 and day +2. Percentages relative to lymphocyte levels measured on day -1 (before FTY720 administration) are plotted over time.

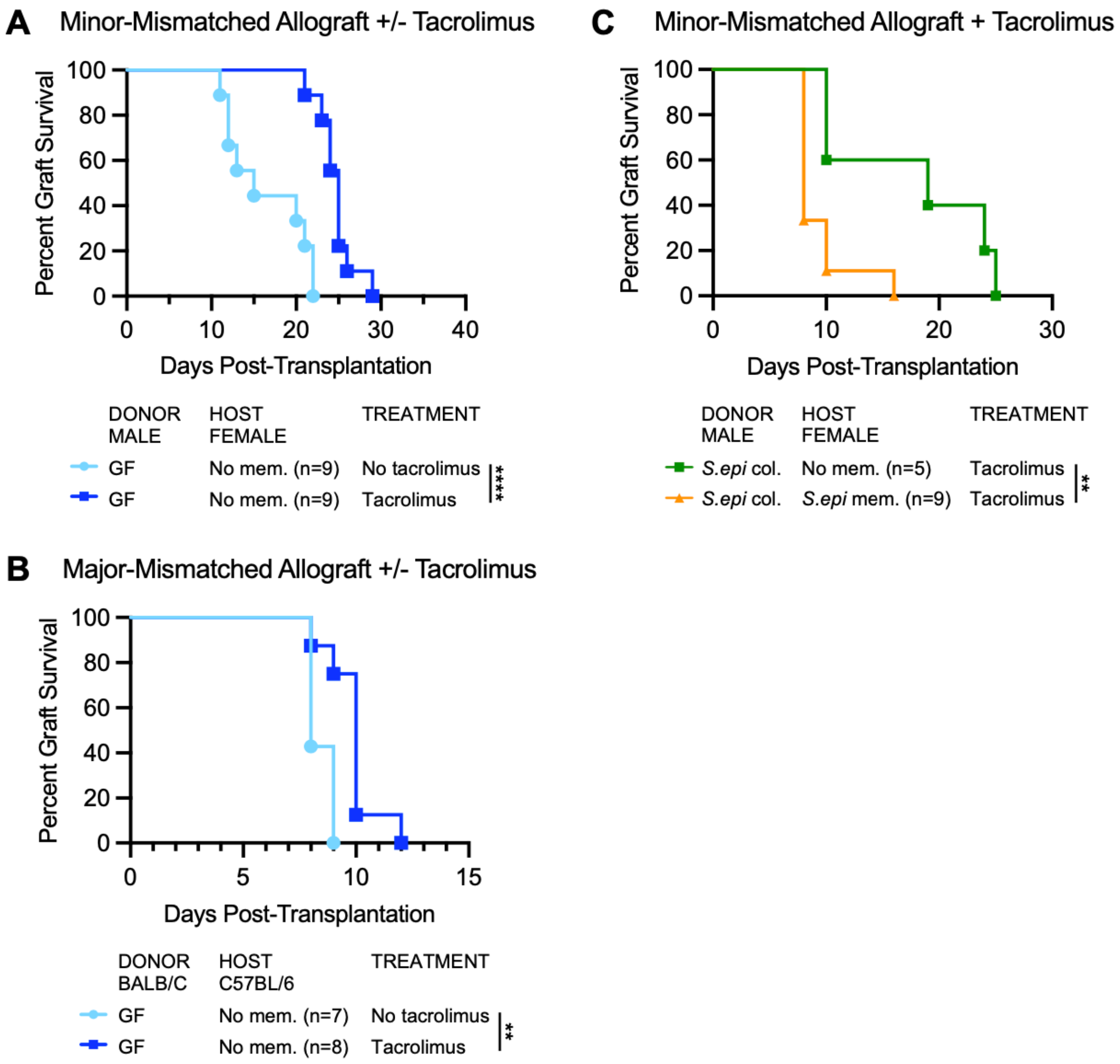

Supplementary Figure 10: Tacrolimus prolongs survival of minor- and major-mismatched skin allografts. (A-B) C57BL/6 females received GF male (A) or BALB/c (B) skin grafts then were treated with tacrolimus (dark blue) or no immunosuppression (light blue). (C) C57BL/6 female hosts with or without anti-*S.epi* memory were transplanted with C57BL/6 male skin grafts colonized with *S.epi*. Hosts received tacrolimus. (A-C) All survival curves were analyzed using a Log-Rank (Mantel-Cox) test. **$p<0.005$, ****$p<0.0001$. mem = memory, col = colonized.

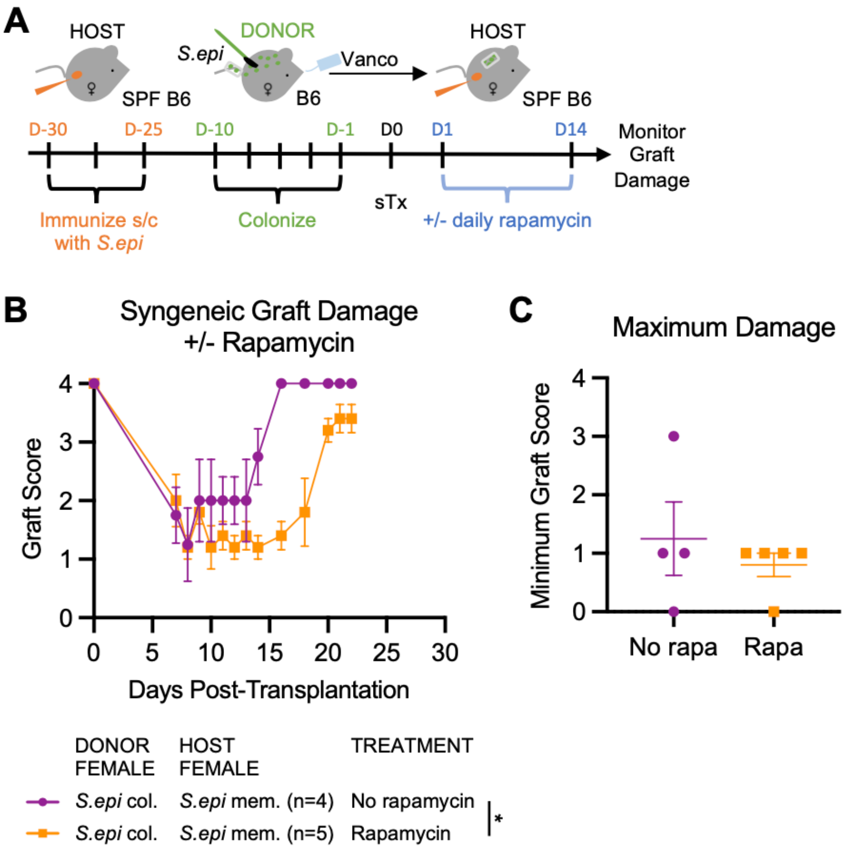

Supplementary Figure 11: Rapamycin does not alleviate the damage sustained by *S.epi*-colonized syngeneic grafts in hosts with anti-*S.epi* memory. (A) C57BL/6 female hosts with anti-*S.epi* memory received *S.epi*-colonized, syngeneic skin grafts and either 30mg/kg/day rapamycin (orange) or no rapamycin (purple) for 14 days after surgery. (B) 4-point graft damage score over time, scored as described in Figure 1D. The AUC was calculated for each individual mouse's graft scores and an unpaired t test was performed on these values *p<0.05. (C) Maximum damage experienced by each graft. Lower score indicates more severe damage. No significant differences were found using an unpaired t test. col. = colonized, mem. = memory.
